# Geospatial Assessments of DNA Adducts in the Human Stomach: A Model of Field Cancerization

**DOI:** 10.3390/cancers13153728

**Published:** 2021-07-24

**Authors:** Yuji Iwashita, Ippei Ohnishi, Yuto Matsushita, Shunsuke Ohtsuka, Takashi Yamashita, Keisuke Inaba, Atsuko Fukazawa, Hideto Ochiai, Keigo Matsumoto, Nobuhito Kurono, Yoshitaka Matsushima, Hiroki Mori, Shioto Suzuki, Shohachi Suzuki, Fumihiko Tanioka, Haruhiko Sugimura

**Affiliations:** 1 Department of Tumor Pathology, Hamamatsu University School of Medicine, 1-20-1 Handayama, Higashi-ku, Hamamatsu, Shizuoka 431-3192, Japan; ippeiohnishi@hospital.iwata.shizuoka.jp (I.O.); yuto.m@hama-med.ac.jp (Y.M.); d15027@hama-med.ac.jp (S.O.); D15025@hama-med.ac.jp (T.Y.); 2 Pathology Division, Iwata City Hospital, 512-3 Ohkubo, Iwata, Shizuoka 438-8550, Japan; shiosuzuki-path@umin.net (S.S.); ft-patho@hospital.iwata.shizuoka.jp (F.T.); 3 Department of Urology, Hamamatsu University School of Medicine, 1-20-1 Handayama, Higashi-ku, Hamamatsu, Shizuoka 431-3192, Japan; 4 Hamamatsu Medical Center, 328 Tomitsuka-cho, Naka-ku, Hamamatsu, Shizuoka 432-8580, Japan; mori_h@hmedc.or.jp; 5 First Department of Surgery, Hamamatsu University School of Medicine, 1-20-1 Handayama, Higashi-ku, Hamamatsu, Shizuoka 431-3192, Japan; 6 Surgery Division, Iwata City Hospital, 512-3 Ohkubo, Iwata, Shizuoka 438-8550, Japan; kinaba@seirei-fuji.com (K.I.); akko@hospital.iwata.shizuoka.jp (A.F.); hydeochi@hmedc.or.jp (H.O.); keigo422@live.jp (K.M.); shohachi88@hotmail.com (S.S.); 7 Department of Chemistry, Hamamatsu University School of Medicine, 1-20-1 Handayama, Higashi-ku, Hamamatsu, Shizuoka 431-3192, Japan; chrono@hama-med.ac.jp; 8 Department of Agricultural Chemistry, Tokyo University of Agriculture, 1-1-1 Sakuragaoka, Setagaya-ku, Tokyo 156-8502, Japan; ym205308@nodai.ac.jp

**Keywords:** DNA adduct, DNA adductome, DNA adductomics, mutagen, exposure, stomach, gastric cancer, liquid chromatography/tandem mass spectrometry

## Abstract

**Simple Summary:**

The geospatial distribution of DNA adducts, the presumable origins of mutations causing human cancers in the stomach was described. In human stomach resections for gastric cancer, seven different DNA adducts, C5-methyl-2′-deoxycytidine, 2′-deoxyinosine, C5-hydroxymethyl-2′-deoxycytidine, N6-methyl-2′-deoxyadenosine, 1,N6-etheno-2′-deoxyadenosine, N6-hydroxymethyl-2′-deoxyadenosine, and C8-oxo-2′-deoxyguanosine, were identified in various parts and zones of the human stomach, namely, the upper, middle and lower zones, anterior and posterior walls, and lesser and greater curvatures. This DNA adductomics approach will reveal the exposure and risk of individual gastric mucosa in humans. Basic information on multiple DNA adduct profiles in terms of the correlation with the preferred locations of gastric cancer in the stomach will provide the reality of “field cancerization” in human gastric carcinogenesis.

**Abstract:**

Background: Field cancerization is a popular concept regarding where cancer cells arise in a plane, such as the opened-up gastrointestinal mucosa. The geospatial distribution of DNA adducts, some of which are believed to initiate mutation, may be a clue to understanding the landscape of the preferred occurrence of gastric cancer in the human stomach, such that the occurrence is much more frequent in the lesser curvature than in the greater curvature. Methods: Seven DNA adducts, C5-methyl-2′-deoxycytidine, 2′-deoxyinosine, C5-hydroxymethyl-2′-deoxycytidine, N6-methyl-2′-deoxyadenosine, 1,N6-etheno-2′-deoxyadenosine, N6-hydroxymethyl-2′-deoxyadenosine, and C8-oxo-2′-deoxyguanosine, from different points and zones of the human stomach were semi quantitatively measured by liquid chromatography/tandem mass spectrometry. The differences in the quantity of these DNA adducts from the lesser and greater curvature, the upper, middle and lower third zones, the anterior and posterior wall of the stomach, and the mucosae distant from and near the tumor were compared to determine whether the location preference of cancer in the stomach could be explained by the distribution of these DNA adducts. Comparisons were conducted considering the tumor locations and operation methods. Conclusions: Regarding the DNA adducts investigated, significant differences in quantities and locations in the whole stomach were not noted; thus, these DNA adducts do not explain the preferential occurrence of cancer in particular locations of the human stomach.

## 1. Introduction

Gastric cancer is the third leading cause of cancer death worldwide [1]. There are regional differences in the incidence of gastric cancer, which occurs frequently in Asia [1]. Although the cause of regional differences is not fully understood, environmental factors are believed to be primarily important in gastric carcinogenesis except in rare cases of a genetic cause [2], mainly because of the time trend of prevalence and immigration studies [3,4,5]. Several chemical carcinogenesis models were developed by Japanese investigators [6,7], but extrapolation of these experimental findings to human stomach carcinogenesis is still elusive. On the other hand, *Helicobacter pylori* (HP) definitely plays an important role in certain steps of gastric carcinogenesis, especially in Japan [8,9], although only a small portion of subjects with HP infections develop gastric cancer [10]. An analysis of human gastric carcinogenesis would provide a model to elucidate the genetic and environmental contributions.

## 2. Gastric Cancer Risk

Several genetic risk loci of gastric cancer are known, ranging from the historically identified blood type A [11] to the markers discovered through genome-wide association study, including prostate stem cell antigen (PSCA), mucin 1 (MUC1) and other genes [12,13,14,15]. As stated in the previous section, immigration studies of Japanese populations to Hawaii or Brazil [3,4,16,17] verified that the main risks for gastric cancer are environmental and lifestyle factors, which include chronic atrophic gastritis [18] and diet, such as salt intake [19]. Recent clean-up of HP has already contributed to decreasing the prevalence of gastric cancer in Japan [9]. Chemical and physical carcinogenesis of the stomach has also been investigated, and several culprits have been proposed, such as talc in refined rice [20]. In particular, based on epidemiological and experimental data, nitroso compounds generated in the stomach are believed to play an important role in human gastric carcinogenesis [21,22,23,24]. Although some of the mechanisms are not completely understood, these risk factors are thought to induce the development of somatic mutations in genes related to carcinogenesis.

## 3. Mutation Spectrum and Mutational Signature of Gastric Cancer

### 3.1. Mutation Spectrum

There are many types of mutations. Even in the case of substitution mutations, three types of substitutions are possible starting from each of the four bases. Based on the complementarity of double-stranded DNA, the combinations can be summarized by six types of substitutions. Mutations observed in individual tumors can be classified into these types. The distribution of these mutation types can be treated as a particular profile, “the mutation spectrum” [25]. If the cause of the mutation is different, the mutation spectrum is also expected to be different. Among the mutations found in gastric cancer, transitions such as C:G to T:A are most prevalent, and such changes in GC and non-GC regions are thought to have different causes. It has been presumed that the C:G to T:A transition that occurs at the CpG site found in gastric cancer originates from the spontaneous deamination of C5-methyl-dC by reactive oxygen species [26,27,28,29]. These mutations in gastric cancer are thought to be caused by inflammation-related carcinogenic mechanisms such as HP. Another pathway to gastric cancer is mutation by alkylating agents generated by nitroso compounds and gastric conditions, in other words, derived from substances present in food and other environments [30,31]. Many of the mutations present in non-GC promoters have been attributed to alkylation of the base. The model of gastric carcinogenesis caused by alkyl compounds has been proposed in Japan using animal experiments [6], but extrapolation to human gastric carcinogenesis has not been fully addressed.

### 3.2. Mutational Signature

The mutation spectrum focuses only on the bases to be substituted, usually in the genes known to be mutated most frequently in human tumors. In the era of massive parallel sequencing, mutational investigation of the comprehensive genome in human stomach cancer expands the horizon of the genes mutated in gastric cancer [32], and the categorization of tumors based on the “mutational signature” has been developed as an analysis approach that also considers the bases before and after the substituted base [33,34]. Early studies applied mutational signatures mostly to gastric cancer exome data and showed that some gastric cancers had mutational signatures specific to repair abnormalities in DNA double-strand breaks, suggesting that the application of platinum therapy or PARP inhibitors may be effective [35]. Although there were certain quantitative differences and biases, some mutational signatures observed in gastric adenocarcinoma were shared by other gastrointestinal adenocarcinomas [36]. Recent pan-cancer analysis of the whole genome showed that gastric adenocarcinoma had several mutational signatures related to the deamination of 5-methylcytosine, APOBEC activity, reactive oxygen species, deficiency of homologous recombination or DNA mismatch repair derived from mutations in BRCA1 or POLD [37]. The prevalence of these mutational signatures and their proportion in the total number of mutations in an individual tumor are different, suggesting different backgrounds for individual instances of carcinogenesis. In general, mutations that appear to be due to loss of DNA repair gene function have relatively low prevalence but represent a relatively high proportion of all mutations in an individual. Some of the mutational signatures are reasoned to reflect the mutational process by distinctive cause, but gastric cancer has a less distinctive signature, although some features are reported [38]. In addition to these mutational signatures, some mutational signatures that show high prevalence within the population and high mutation rates in individual tumors have unknown causes. Intestinal metaplasia, usually distinct from authentic cancer, is known to have a low frequency of DNA mutations, and its mutational signature partially overlaps with the mutational signature of adjacent full-blown gastric cancer [39]. Most of the cancer genomic data to date have been biased towards individuals of European descent, but studies of the cancer genome in Africans and Asians are becoming increasingly important. Asians are known to have a high incidence of gastric cancer, and elucidation of the reason is a challenge. For example, regarding the association with HP, which is one of the important causes of gastric cancer, Shimizu et al. reported a specific mutation spectrum by the analysis of a small number of the cases associated with HP [40]. Recent analysis using hundreds of Japanese gastric cancer exomes discovered that a particular mutational signature is associated only with people who have both smoking and drinking habits [41]. Mutational signature analysis is expected to identify new causes of gastric carcinogenesis, but not all hypotheses about the causes of mutations derived from mutation spectrum analysis studies have been verified by current mutational signature analysis. There should be other molecular clues to focus on the ultimate cause of DNA damage and mutations.

## 4. DNA Adducts as a Cause of Mutation

### 4.1. DNA Damage in Gastric Mucosae

#### 4.1.1. Types of DNA Damage

**Figure 1:**
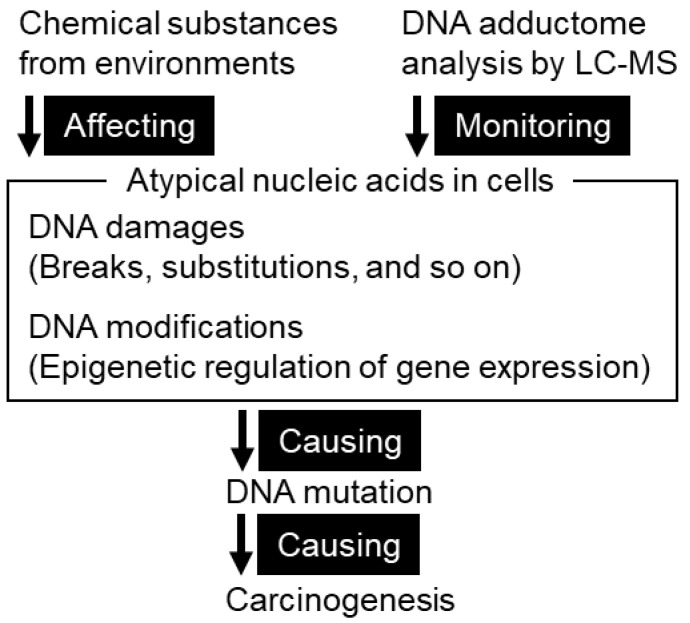
A schematic illustration of DNA adductome analysis to monitor DNA damages in chemical carcinogenesis. Many chemical substances affect DNA damages and DNA modifications. These atypical nucleic acids potentially cause DNA mutation and carcinogenesis. Using liquid chromatography coupled with mass spectrometry, the accumulation of DNA damages can be monitored by DNA adductome analysis.

The gastric mucosa, like other organs, is subject to various types of DNA damage, including DNA single- or double-strand breaks and the formation of DNA adducts, by highly reactive chemicals generated externally or internally. This review focuses on DNA adducts that can cause DNA mutations in chemical carcinogenesis (Figure 1). DNA adducts are produced due to chemical damage caused by highly reactive substances derived from food and the environment or substances that exhibit increased reactivity after metabolic processes inside and outside the body. The products of enzymatic DNA modifications associated with epigenetic regulation, such as C5-methyl-dC, have chemical characteristics in common with DNA adducts in the sense of altered atypical nucleic acids. Depending on the chemical structure, the DNA adduct may cause incorrect base pairing or interfere with DNA polymerase during DNA replication. These factors inhibit accurate DNA replication and potentially cause DNA mutations. Regardless of mutagenicity, DNA adducts in cells are useful as indicators of exposure to factors associated with carcinogenesis. In addition, they are used to study the etiology of mutations as a result of chemical reactions that occur before mutations associated with carcinogenesis.

#### 4.1.2. Methodology for Identification of DNA Adducts

Historically, the ^32^P postlabeling method has been widely used as a method for detecting DNA adducts [42]. Purified DNA is digested to monomeric nucleotides by enzymes and postlabeled by T4 polynucleotide kinase and [γ-^32^P] ATP and then separated by thin-layer chromatography to distinguish DNA adducts from intact nucleobases that are present in large excess in cells. The ^32^P postlabeling method is highly sensitive and is estimated to detect only one DNA adduct in the 10^10^ undamaged bases. However, the identification of the type of DNA adducts based on the results of the standard compounds used in parallel was ambiguous. Subsequently, a method was developed in which a single DNA adduct was targeted, well separated by high-performance liquid chromatography (HPLC), and identified by ultraviolet absorption or mass spectrometry. With a few exceptions, both the mass and the column retention time of each DNA adduct often vary, greatly improving the accuracy of substance identification. With the further development of methods for comprehensively identifying or quantifying intracellular molecules by mass spectrometry, such as proteomics and metabolomics, the DNA adductomics approach has also evolved [43,44].

#### 4.1.3. DNA Adductome

A set of all DNA adducts present in cells is defined as a DNA adductome. The academic discipline or methodology that deals with the DNA adductome is called DNA adductomics. The advantage of performing liquid chromatography coupled with mass spectrometry as a DNA adductomics approach is to identify many types of DNA adducts with a degree of certainty and quantify them at the same time. The similarity to disciplines such as proteomics and metabolomics is that mass spectrometry is used to identify and quantify the relevant biomolecules as completely as possible. An important point common to these comprehensive analyses is the justification of peak determination. If available, standard compounds can be primarily used to identify the m/z, that is, the mass per number of charges observed in the mass spectrometry of DNA adducts. The standards are also useful to determine peak boundaries in LC-MS chromatograms, helping to distinguish between signal and noise. Information on the chemical standards of DNA adducts can be collected from research articles and reviews [45,46,47,48] and public or commercial databases such as PubChem and SciFinder. An international consortium deposited the mass spectrum of DNA adducts to a mass database [49]. Many chemical vendors respond to inquiries about compound synthesis. In the absence of standards, the signal intensity and frequency within the population are factors to consider when selecting peaks to be measured as potential DNA adducts. As the calculation of the peak area is affected by the drift of the column retention time, an appropriate signal threshold is also an issue. Signal normalization between samples is necessary because there are variations in measurement within the same day or between days.

DNA adductomics has several unique characteristics as an analytical experiment. Although a simple comparison is not possible, there are four types of intact nucleobases in the human genome and approximately 20 types of amino acids that constitute peptides, whereas there are over 200 types of DNA adducts with unique chemical structures derived from various mutagens [50]. Therefore, it is necessary to address the different degree of complexity from that of DNA sequencing and protein identification. In the multiple processes for the identification of DNA adducts, including DNA purification, DNA digestion and preprocessing for mass spectrometry, such as enzyme removal and concentration, effective methods vary depending on the chemical properties of each individual DNA adduct. The variables to consider include the material of the sample vial and the composition of the sample solvent. Optimal storage may vary because the chemical diversity of DNA adducts also affects their stability. Although it is a basic concept in scientific research, the need to process the sample groups to be compared in parallel and control the artificial variation is exceptionally strong. Signals from excess genomic DNA can interfere with the identification of DNA adducts based on m/z and column retention time. However, naturally occurring isotopes from these intact deoxyribonucleosides are available for the normalization for the intraday and day-to-day variance in mass spectrometric measurements. Iatrogenic, artificial and other confounding factors, especially in human tissues, forced us to modify the DNA extraction protocol.

The data analysis of the DNA adductome is also unique. In general, DNA adducts have low abundance and low frequency. The distribution of the abundance of each DNA adduct in the population, such as a normal distribution or a Poisson distribution, is not yet fully understood. The optimization of statistical frameworks such as parametric or nonparametric methods in accordance with such situations is still an unsolved problem. The low abundance of DNA adducts raises the question of whether to include measured zero values in statistical tests, but no consensus has yet been formed. It is useful to perform both qualitative and quantitative analyses with transparency. Sensitivity management is required for qualitative analysis because its detection or the lack thereof alone may be meaningless due to variations in sensitivity between specimens and measurement days. On the other hand, avoiding normalization between specimens provides an advantage in qualitative analysis. Common DNA adducts that are detected in all samples, such as C5-methyl-dC and C5-hydroxymethyl-dC, cannot be qualitatively analyzed. Qualitative analysis is appropriate for DNA adducts that are expected to occur infrequently due to exposure.

### 4.2. DNA Adduct Profile of Gastric Mucosae

#### 4.2.1. Lipid Peroxidation-Induced DNA Adducts in Human Gastric Mucosa

We applied a DNA adductomics method to DNA extracted from 22 gastric mucosae of Japanese and Chinese individuals and observed seven DNA adducts related to lipid peroxidation: 1,N6-etheno-2′-deoxyadenosine, butanone-etheno-2′-deoxycytidine, butanone-etheno-2′-deoxy-5-methylcytidine, butanone-etheno-2′-deoxyadenosine, heptanone-etheno-2′-deoxycytidine, heptanone-etheno-2′-deoxyadenosine and heptanone-etheno-2′-deoxyguanosine [51]. These DNA adducts showed different abundances ranging from below the detection limit to 1 adduct/10^5^ corresponding nucleobases in individuals. Although there was no clear association of these DNA adducts with HP infection or other clinicopathological information, the accumulation of these lipid peroxidation-related DNA adducts was different between the Japanese and Chinese gastric mucosae. The population-dependent differences in the abundance of DNA adducts suggest that the DNA adductome profile may reflect the genetic or environmental factors that characterize each population. Our evaluation also revealed the repair mechanism of these lipid peroxidation-related DNA adducts [52]. Examining eight DNA glycosylases, including *OGG1*, *SMUG1*, *TDG*, *NEIL1*, *MUTYH*, *NTH1*, *MPG* and *UNG2*, we confirmed the results of previous studies in which SUMG1 and MPG removed 3,N4-etheno-2′-deoxycytidine and 1,N6-etheno-2′-deoxyadenosine, respectively [53,54]. The 3,N4-etheno-2′-deoxycytidine is another DNA adduct induced by lipid peroxidation. We also found that TDG was able to remove thymine that has mispaired with several lipid peroxidation-related DNA adducts. Mass spectrometric analysis showed that the global amount of 3,N4-etheno-2′-deoxycytidine was increased by knockdown of TDG protein in cultured cells. As part of the diverse DNA damage that occurs in the human gastric mucosa, DNA adducts may create population- or individual-specific carcinogenic conditions through a complex process involving DNA repair mechanisms.

#### 4.2.2. Application of DNA Adductomics Technology to the Analysis of Cytosine Modifications in Gastric Cancer

DNA adductomics technology is applicable to enzymatically modified DNA. C5-methyl-2′-deoxycytidine is one of the most abundant DNA modifications and plays a pivotal role in the epigenetic regulation of gene expression [55]. This modified base is not considered a cause of DNA mutations associated with disturbed DNA replication. Since its chemical structure and properties are different from those of undamaged nucleobases, it can be measured by mass spectrometry, similar to other mutagenic DNA adducts. C5-hydroxymethyl-2′-deoxycytidine, C5-formyl-2′-deoxycytidine and C5-carboxyl-2′-deoxycytidine are produced by the oxidation of methylated cytosine by multiple enzymes as a demethylation process. We performed DNA adductome analysis in human gastric mucosae to quantify these DNA methylation or demethylation-related atypical nucleobases [56]. C5-hydroxymethyl-2′-deoxycytidine was significantly decreased in the tumorous portion of gastric cancer. C5-methyl-2′-deoxycytidine was also moderately decreased in tumors, while C5-formyl-2′-deoxycytidine and C5-carboxyl-2′-deoxycytidine were barely detectable. The expression of several enzymes related to cytosine demethylation was notably decreased in gastric cancer compared with that in adjacent nontumor regions. TET1 expression and C5-hydroxymethyl-2′-deoxycytidine levels had a significantly positive correlation. TET1 had a greater effect on the increase in C5-hydroxymethyl-2′-deoxycytidine in a cultured cell line. The altered amount of modified bases regulated by the activity of methylation and demethylation enzymes is considered to be one aspect of biomolecular transformation in tumors.

#### 4.2.3. Mass Spectrometric Profiling of DNA Adducts in the Human Stomach Associated with Damage from Environmental Factors

To further explore the DNA adducts observed in the human stomach, we analyzed gastric mucosae resected from gastric cancer or non-gastric cancer patients using wide-targeted identification of DNA adducts by liquid chromatography coupled with mass spectrometry [57]. We identified seven DNA adducts including modified bases, C5-methyl-2′-deoxycytidine, 2′-deoxyinosine, C5-hydroxymethyl-2′-deoxycytidine, N6-methyl-2′-deoxyadenosine, 1,N6-etheno-2′-deoxyadenosine, N6-hydroxymethyl-2′-deoxyadenosine, and C8-oxo-2′-deoxyguanosine, in the human stomach. N6-hydroxymethyl-2′-deoxyadenosine was first observed in the human gastric mucosa and follows recent reports of its presence in tumor and nontumor portions of human lung tissue [58]. A comparison of multiple samples in each individual showed that the differences within the individual were small, but the differences between the individuals were large. The causes of individual differences are not fully understood, but some may reflect differences in genetic background and/or exposures that vary from individual to individual. In fact, individuals with a history of smoking and/or drinking had a high accumulation of 1,N6-etheno-2′-deoxyadenosine. C5-hydroxymethyl-dC was high in the gastric mucosa of non-gastric cancer patients, low in the nontumor regions of gastric cancer patients, and even lower in the tumor regions. It is noteworthy that changes in the nontumor part of the gastric mucosa may be a precursor to carcinogenesis. There are certain correlations between the accumulation of different DNA adducts, and it is expected that DNA adducts are generated through multiple pathways. By accumulating these discoveries, we are evaluating whether the profile of the DNA adductome, along with DNA mutations and gene expression, will be important information for understanding the mechanism of carcinogenesis.

## 5. Field Cancerization and DNA Adductomics in the Stomach

### 5.1. Pathology of Gastric Cancer

#### 5.1.1. Practice in Gastric Cancer Management: Detection, Diagnosis, and Therapy

The practice of gastric cancer management is different among countries depending on prevalence, detection system, and surgical management. Early detection is the norm in some countries, such as Japan, but in most countries, the targets of medical care are advanced-stage patients. Most gastric cancer is adenocarcinoma. The morphology of adenocarcinoma of the stomach has attracted many pathologists, and subtyping based on histopathology and morphology is still popular, from the dichotomy of intestinal vs. diffuse types in Laurén’s classification [59] to classifications including papillary type [60,61,62] and other minor subtypes, such as chief-cell predominant type [63] and fetal-gut differentiation type [64]. Morphological recordings of early-stage gastric cancers are summarized in the Japanese Classification System [65], and the system has fundamentally influenced the WHO classification system [60] since the first edition [66]. Guidelines for therapies, from surgery to chemotherapy to molecular targeted or immune checkpoint inhibitor therapy, are proposed almost annually [67]. Molecular-level advances in terms of infection, intestinal metaplasia, oxidative stress as an initiating cause, and progression model are also detailed [68].

#### 5.1.2. Recent Molecular Characterization of Gastric Cancer

The somatic driver mutations that cause carcinogenesis are thought to be acquired in each gastric cancer showing various pathological conditions. The classical method of classifying gastric cancer by acquired somatic mutations started with mutations in *TP53* and *CDH1* and expanded the target driver genes through systematic exome or whole-genome sequencing. Beginning with the discovery that mutations in the ARID1A [69] and RHOA [70,71] genes are frequently found in subgroups of gastric cancer, a comprehensive molecular classification of gastric cancer was performed in hundreds of people, subdividing gastric cancer into four types: Epstein-Barr virus-positive, microsatellite instable, genomically stable and chromosomally instable tumors [72]. Research on whole-genome analysis began with a small number of cases [32] and has now grown to the scale of hundreds [73], and it is ultimately becoming possible to classify driver genes of individual cases of gastric cancer. As mentioned above, classification using mutational signature analysis of gastric cancer is also actively performed [37]. The identification in tumors of driver mutations that caused carcinogenesis enables us to evaluate the relationship between each subtype and prognosis and the effectiveness of various treatment methods, for example, by the elucidation of the genes that define the response to preoperative chemotherapy [74]. Moreover, the information on molecular evolution and the heterogeneity of peritoneal carcinomatosis derived from gastric adenocarcinoma [75] and the association of epidemiologic and clinical risk factors with each subtype are current topics that many molecular biologists have reviewed extensively [76,77,78,79].

### 5.2. Field Cancerization

#### 5.2.1. Original Concept of Field Cancerization

The concept of field cancerization was proposed by Slaughter in 1953 [80], taking oral squamous cell carcinoma as an example. The planes composing stratified squamous epithelium such as oral, esophageal and skin tissue are good examples of this model; the point in the plane with the highest exposure to carcinogenic risk undergoes the initiation of carcinogenesis: that is, when several areas or sites are occupied by cancers, multiple occurrences would be a better interpretation of this phenomenon than horizontal invasion by one group of tumor cells. The supposed density map of carcinogens would influence the selection of the point most vulnerable to carcinogenic effects in the plane of the mucosa or skin. Molecular events such as the spread of the *TP53* mutation in the skin reflect field cancerization in molecular terms [81,82]. In addition to mutations, epigenetic changes in the field also provide important information on earlier changes in DNA, such as promoter methylation in “precancerous” and apparently unremarkable mucosa [83]. Mitochondrial DNA mutations [84], mutation burdens [85] and the expression of miRNAs [86] are also relevant to the field cancerization hypothesis. Recent deep sequencing in the “normal” area of the tissues found considerable evidence of mutations [87], some of which are clonal. These observations supported the spatial occurrence of mutation and evolutionary processes in the origin and development of human cancer [88]. The density of attributes that include carcinogens themselves, epigenetic lesions, microRNA and mitochondrial DNA mutations and various DNA mutations found in precancerous or apparently nontumor mucosa on the planes of the mucosa will reflect the preference of cancer occurrence in the gastrointestinal tract in terms of spatial locations. The significance of multisite sampling for ultrarare somatic mutations in noncancerous tissue in field cancerization theory is well recognized, but the profile of these mutations has not been fully linked to the profile of DNA adducts in situ as an initiation step of individual mutations.

#### 5.2.2. Field Cancerization of Gastric Mucosae and Preferential Cancer Location within the Stomach

Gastric cancer is known to occur relatively often on the lesser curvature side or fundus [89,90,91]. Although it is hypothesized that the lesser curvature side is a path for food and is susceptible to various stimuli and damage, the cause of hot spots is not fully elucidated. It is also known that the characteristics of gastric cancer that occur in the cardia or non-cardia are different [92,93,94,95]. These matters related to field cancerization are also considered to be related to the recurrence of gastric cancer and the development of multiple tumors. To elucidate the topography and contiguous pathology of gastric cancer, multisite sampling is important. Although there is limited research on the altered abundance of biomolecules in gastric mucosae, the molecules and phenomena observed in different abundances in the gastric mucosa are diverse, including DNA mutations and the expression of genes, microRNAs [86,96] and circular RNAs [97]. The accumulation of genetic and epigenetic alterations in normal cells and consequent cancer risk have also been reported [83,98,99].

#### 5.2.3. Mass Spectrometric Profiling of DNA Adducts in the Local Part of the Human Stomach

**Figure 2:**
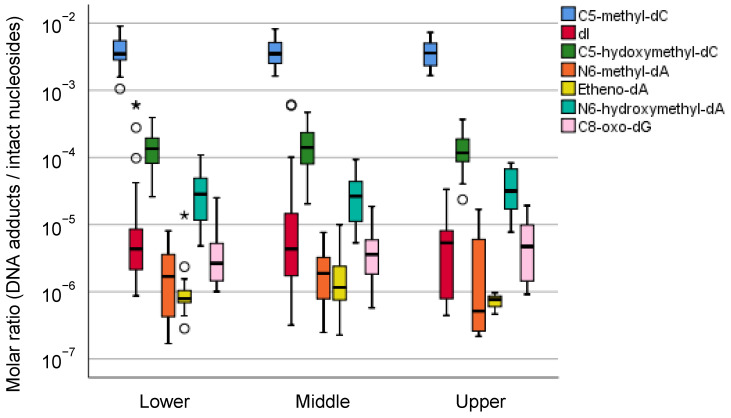
Distribution of DNA adduct profiles in the upper, middle, and lower parts of the gastric mucosae of gastric cancer patients. The molar ratio data of DNA adducts were retrieved from our previously published research [57]. The center line of the box indicates the median, and the top and bottom edges of the box indicate their interquartile ranges (IQR). The whiskers show the maximum or minimum values within a range of 1.5 times the IQR from the edge of the box. Outliers are indicated by circles (°) that are 1.5 to 3 times the IQR from the top or the bottom of a box and by stars (*) that are more than 3 times the IQR from the top or the bottom of a box.

**Figure 3:**
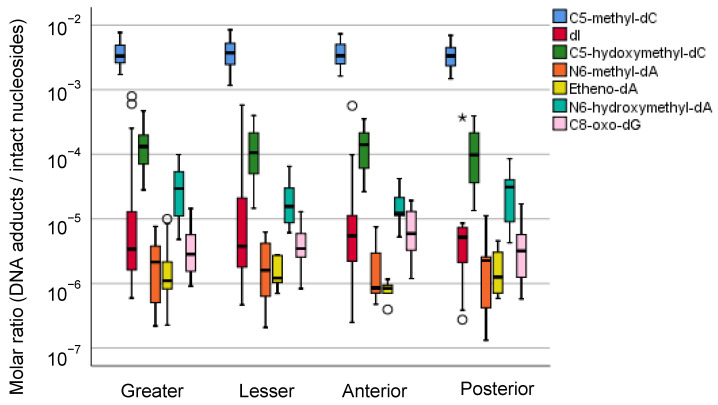
Distribution of DNA adduct profiles in the greater curvature, lesser curvature, anterior wall, and posterior wall of the gastric mucosae of gastric cancer patients. The molar ratio data of DNA adducts were retrieved from our previously published research [57]. The center line of the box indicates the median, and the top and bottom edges of the box indicate their interquartile ranges (IQR). The whiskers show the maximum or minimum values within a range of 1.5 times the IQR from the edge of the box. Outliers are indicated by circles (°) that are 1.5 to 3 times the IQR from the top or the bottom of a box and by stars (*) that are more than 3 times the IQR from the top or the bottom of a box.

As described above, we have reported an interindividual variance in the accumulation of DNA adducts in human gastric mucosae [57]. Although we also showed that the intraindividual difference in DNA adducts was small, we reanalyzed all the available data according to their anatomical information. Seven DNA adducts observed in human gastric mucosae showed a wide range of molar ratios (1 DNA adduct/10^6^–10^2^ intact nucleosides), but there was no difference in the anatomical zone-specific accumulation of these DNA adducts (Figure 2 and Figure 3). As alternative approaches to verify the association between the DNA adduct and gastric carcinogenesis, we are considering analyses limited to typical samples according to the location of the tumor or length from the tumor.

## 6. Perspectives to Evaluate the Field Cancerization of Gastric Cancer

### 6.1. Mutational Signature Analysis Combined with Experimental Exposure and Perturbation of Genetic Backgrounds

The combination of new technologies is expected to accelerate the verification of the field cancerization hypothesis. Mutational signatures in genomics have begun to be cataloged in association with experimental exposure to environmental mutagens [100], which not only provides a history of exposures received by individuals but can also depict the situation of local exposure for each specimen. Similar studies have been conducted in mice, arguing that exposure to various chemical carcinogens does not produce fully unique mutational signatures, but only specific mutational signatures are observed [101]. Both the estimation of mutational signatures by deep sequencing and the direct identification of DNA adducts by mass spectrometry reveal the chemical changes that DNA has undergone, but only a few studies have combined the two methods [102,103,104,105]. It becomes important to understand the state before and after the occurrence of mutation by these two methods based on different principles. The consideration of the genetic background in mutagenesis using experimental exposure is also important. A pioneering study using *C. elegans* revealed mutational signatures after 20 generations of propagation or exposure to carcinogens in the background of deletions of 17 DNA repair genes related to nucleotide excision repair, base excision repair, DNA crosslink repair, DNA end-joining and apoptosis [106]. Mutational signatures induced by chemical carcinogens are augmented with a unique tendency by the deletion of specific DNA repair genes, indicating that both genetic background and the type of mutagen are important to elucidate the cause of the mutation. They extended their strategy to the deletion of mismatched repair genes such as *MLH1* and achieved increased numbers of mutations with only 20 generations of propagation. This may mimic the condition of carcinogenic mutations in gastric cancer with microsatellite instability [107]. One of the three mismatch repair-related mutational signatures obtained by combining exomes of human gastric and colorectal cancer was consistent with that found in the nematode experiment. This has shown some of the usefulness of connecting the causes and results of mutation in experiments with model animals, but it is also true that mutational signatures of unknown etiology derived from human tumors remain. In a recent study with deletions of 53 DNA repair genes and exposures to 12 genotoxins, including ultraviolet B rays, ionizing radiation, alkylating compounds, aristolochic acid, aflatoxin B1 and cisplatin, 41% of experiments showed changes in the total number of mutations or mutational signatures [108]. In another cutting-edge study, knockouts of human-induced pluripotent stem cells in which 42 DNA repair or DNA replication genes were deleted by CRISPR-Cas9 underwent whole-genome sequencing to profile somatic mutations and understand mutation mechanisms that were previously unclear [109].

### 6.2. Detection of DNA Damage by DNA Sequencing

In the current mutational signature analysis by deep sequencing, in principle, it is not possible to know where DNA damage, including DNA adducts, was located on the genome before the formation of mutations. The genomic site of DNA damage can be determined through the purification of damaged DNA and deep sequencing. There are various methods for purifying damaged DNA, such as XR-seq [110,111], which uses an antibody against TFIIH to collect oligonucleotides generated in the process of excision repair, and Damage-seq [110], which uses an antibody against damaged DNA such as cisplatin and ultraviolet photoproducts. The sequencing of bulky DNA adducts that inhibit DNA amplification required for deep sequencing was improved using translesion DNA synthesis polymerase as tXR-seq [112]. These methodologies were applied to evaluate drug resistance in oxaliplatin treatment [113] and transcription-coupled repair in ribosomal DNA [114]. For several mouse tissues, the integration of the sequencing of damaged DNAs and classical genome-wide characteristics derived from RNA-seq and ChIP-seq enabled us to reveal unprecedented genomic aspects of DNA damage influenced by gene expression and chromatin state [115]. As a different principle, single molecule real-time sequencing by a sequencer from Pacific Biosciences can also be used to sequence C5-methyl-2′-deoxycytidine and N6-methyl-2′-deoxyadenosine at 1-base resolution [116]. This method has been shown to be applicable to other damaged DNAs, such as C8-oxo-2′-deoxyguanosine, C8-oxo-2′deoxyadeosine, O6-methyl-2′-deoxyguanosine, N1-methyl-2′-deoxyadenosine, O4-methyl-2′-deoxythymidine, C5-hydroxy-2′-deoxycytidine, C5-hydroxy-2′-deoxyuridine, and C5-hydroxymethyl-2′-deoxyuridine or thymine dimers [117]. As another alternative method, it has been reported that the positions and sequences of thymidine analogs [118] and alkylated DNA [119] are determined by nanopore sequencing using MinION from Oxford Nanopore Technologies (Oxford, UK).

### 6.3. Social and Scientific Significance of the Field Cancerization of Gastric Cancer

Since these DNA sequencing-based methods limit the number of DNA adducts that can be analyzed at the same time, they are effective when used to complement the methods using mass spectrometry. To apply these techniques to verify the field cancerization hypothesis, it is necessary to collect multiple samples in continuous fields of tissues, but so far, examples of verifying local differences in DNA damage of the human gastric mucosa within one individual are still few. The regional specificity of DNA damage may be responsible for local carcinogenesis of gastric mucosae. After the further development of these technologies, it will be possible to catalog molecular types of DNA damage and their causative agents as clinical data through laboratory tests. These data help to control exposure and prevent carcinogenesis. In addition to the early detection of cancer and precise treatment specific to the individual, establishing a lifestyle that is less likely to cause cancer due to less DNA damage has important public health implications.

In this report, we present the spatial distribution of the seven DNA modifications in the stomach. These DNA modifications may be secondary products of chronic inflammation and continued exposure to reactive oxygen species. As far as these seven DNA modifications are concerned, no particular preference of the distribution was noted (Figure 2 and Figure 3). According to Sasazuki et al. [120], smoking was associated with an increased risk of the differentiated type of distal gastric cancer; compared to the group who never smoked, the adjusted relative ratios (RRs) of gastric cancer for past and current smokers were 2.0 (95% confidence interval = 1.1–3.7) and 2.1 (95% confidence interval = 1.2–3.6), respectively. No association was observed between cigarette smoking and the risk of the undifferentiated type of distal gastric cancer except for a suggestive association with cardia cancer. For alcohol consumption, elevated risk was suggested only for cardia cancer of all histologic types, although the relationship failed to reach significance. Further analysis is needed for large-scale epidemiological observations, and particular DNA adducts in gastric cancer in particular locations may be related to some lifestyle factors. The differences seem to be more dependent on individual exposure to tobacco and alcohol, which are known to contribute to stomach cancer to some degree. The compound effects of several lifestyle factors ranging from educational records to diet were examined in a Japanese Public Health Cohort study [121]. Of course, there are other categories of DNA adducts or modifications that reflect the preferential distributions of cancer occurrence and are probably more related to the mechanistic injury of DNAs of the stomach causing subsequent mutations.

## 7. Conclusions

Although a peculiar preference among the locations in the stomach has been known, the precancerous changes at the molecular level in the landscape of the gastric mucosa did not explain the occurrence of gastric cancer. We showed a spatial adductomics approach as a possible approach to zero in on the local spot of the stomach for the initiation of cancer, but the findings presented here do not cover several bulky DNA adducts that are more familiar in experimental animal models. An extensive search for DNA adduct distribution in the stomach with mutational signatures caused by the initiating changes will help to produce a realistic view of human carcinogenesis and some preventive procedures that will become feasible in the future.

## Data Availability

The data presented in this study are available on request from the corresponding author.
